# Germinated Brown Rice Alters A*β*(1-42) Aggregation and Modulates Alzheimer's Disease-Related Genes in Differentiated Human SH-SY5Y Cells

**DOI:** 10.1155/2015/153684

**Published:** 2015-12-22

**Authors:** Nur Hanisah Azmi, Maznah Ismail, Norsharina Ismail, Mustapha Umar Imam, Noorjahan Banu Mohammed Alitheen, Maizaton Atmadini Abdullah

**Affiliations:** ^1^Nutricosmeceuticals and Nutrigenomics Programme, Laboratory of Molecular Biomedicine, Institute of Bioscience, Universiti Putra Malaysia, 43400 Serdang, Selangor, Malaysia; ^2^Department of Nutrition and Dietetics, Faculty of Medicine and Health Sciences, Universiti Putra Malaysia, 43400 Serdang, Selangor, Malaysia; ^3^Department of Cell and Molecular Biology, Faculty of Biotechnology and Biomolecular Sciences, Universiti Putra Malaysia, 43400 Serdang, Selangor, Malaysia; ^4^Department of Pathology, Faculty of Medicine and Health Sciences, Universiti Putra Malaysia, 43400 Serdang, Selangor, Malaysia

## Abstract

The pathogenesis of Alzheimer's disease involves complex etiological factors, of which the deposition of beta-amyloid (A*β*) protein and oxidative stress have been strongly implicated. We explored the effects of H_2_O_2_, which is a precursor for highly reactive hydroxyl radicals, on neurotoxicity and genes related to AD on neuronal cells. Candidate bioactive compounds responsible for the effects were quantified using HPLC-DAD. Additionally, the effects of germinated brown rice (GBR) on the morphology of A*β*(1-42) were assessed by Transmission Electron Microscopy and its regulatory effects on gene expressions were explored. The results showed that GBR extract had several phenolic compounds and *γ*-oryzanol and altered the structure of A*β*(1-42) suggesting an antiamyloidogenic effect. GBR was also able to attenuate the oxidative effects of H_2_O_2_ as implied by reduced LDH release and intracellular ROS generation. Furthermore, gene expression analyses showed that the neuroprotective effects of GBR were partly mediated through transcriptional regulation of multiple genes including Presenilins, APP, BACE1, BACE2, ADAM10, Neprilysin, and LRP1. Our findings showed that GBR exhibited neuroprotective properties via transcriptional regulation of APP metabolism with potential impact on A*β* aggregation. These findings can have important implications for the management of neurodegenerative diseases like AD and are worth exploring further.

## 1. Background

Alzheimer's disease (AD) is a progressive disorder of the central nervous system, with huge economic burden to individuals and their societies [1]. AD progression is accompanied by gradual loss of neurons responsible for learning and memory. Beta-amyloid (A*β*) plaques are often found deposited in the brain of AD patients and may be responsible for the loss of synaptic transmission and neuronal death that are characteristic of AD [2]. Even though AD has a complex pathogenesis and its etiology is not precisely understood, major findings have suggested that dysregulation of amyloid precursor protein (APP) metabolism plays a major role in the generation of neurotoxic A*β* [2]. Accordingly, differential regulation of APP metabolic processing involving *α*-, *β*-, or *γ*-secretases will result in either amyloidogenic or nonamyloidogenic products with huge implications for disease outcomes [3].

In normal physiologic condition, APP is cleaved by *α*-secretase to produce the nonamyloidogenic soluble neurotrophic fragment (sAPP*α*), thereby precluding the formation of the toxic A*β* [4]. On the other hand, cleavage of APP by *β*-secretase and *γ*-secretase follows the amyloidogenic pathway, thus releasing A*β* peptides into the extracellular matrix [5]. The *β*-secretase components include A*β* cleaving enzymes 1 and 2 (BACE1 and BACE2, resp.) while *γ*-secretase is formed by Presenilin, nicastrin, Aph-1, and Pen-2 [5–7]. Despite clear evidence that inherited mutations of APP and Presenilin genes are causal factors for early onset familial AD [8, 9], the exact mechanism underlying overproduction of A*β* in late onset sporadic AD is not known, even though sporadic AD accounts for the majority of AD cases.

As can be recalled, oxidative stress in the brain often results from inability of endogenous antioxidant defense to counter the reactive oxygen species (ROS) produced by the brain cells. Experimental models had demonstrated the JNK-dependent upregulation of BACE1 and *γ*-secretase activity as a result of oxidative stress [10] in neuroblastoma cells. Similarly, PC12 cells induced by hydrogen peroxide (H_2_O_2_) significantly increased BACE1-promoter activity and augmented A*β* production from APP carrying the human Swedish mutation [11]. Furthermore, disruption of APP metabolism has been demonstrated in oxidative stress [10, 11], while increased levels of BACE1 have been reported in the brain region of sporadic AD patients [12]. These findings are further supported by the links between oxidative stress, altered mitochondrial function, and dysregulation of calcium hemostasis that precede A*β* plaques development with AD neuropathology [13, 14].

It has been proposed that antioxidants have huge potentials in preventing and/or delaying the development and progression of neurodegenerative diseases including AD [15]. This has driven the search for potent antioxidants with neuroprotective potentials. Germinated brown rice (GBR) has been reported to possess important biological activities including antioxidative and neuroprotective properties [16, 17]. Additionally, it was reported to protect neuronal cells from oxidative stress-induced cytotoxicity, alleviate depression-like behaviors as well as enhance learning ability, and improve memory impairment resulting from A*β* administration [18–21]. These properties of GBR are thought to be mediated by its bioactive-rich constituents [22]. At present, there is limited knowledge on the effects of oxidative stress on normally expressed APP. Nonetheless, it is suggested to represent a near-physiological model of neuronal A*β* generation [10]. Since H_2_O_2_ is the main source of the highly reactive hydroxyl radical in the brain, in the present work we studied its effects on the levels of and processing of human APP by *α*-, *β*-, and *γ*-secretase and the role of the low density lipoprotein receptor-related protein (LRP) and Neprilysin, as well as the potential of GBR to attenuate such processes. This study is also the first to explore the modulation of APP metabolism in differentiated human neuroblastoma cells by GBR as well as its effect on the aggregation of A*β in vitro*.

## 2. Materials and Methods

### 2.1. Materials and Chemicals

Brown rice (BR) of Malaysian mixed varieties (MR219 and MR220) was procured from Padiberas Nasional (BERNAS) factory, Seri Tiram Jaya, Selangor. Food grade H_2_O_2_ and sodium hypochlorite (NaOCl) were purchased from Bendosen Laboratory Chemicals (Selangor, Malaysia) and from Dexchem Industries Sdn. Bhd. (Penang, Malaysia), respectively. Human SH-SY5Y neuroblastoma cell line was purchased from the American Type Culture Collection (ATCC), USA. Dulbecco's Modified Eagle's Medium/Nutrient F-12 mixture, fetal bovine serum, gentamicin, phosphate-buffered saline (PBS), human A*β*(1-42), 2,7-dichlorodihydrofluorescein diacetate (DCFH-DA), and trypsin were purchased from Sigma-Aldrich (St. Louis, MO, USA). Analytical grade H_2_O_2_ was purchased from Merck (Darmstadt, Germany), while CytoTox96 Non-Radioactive Cytotoxicity Assay was purchased from Promega (Southampton, UK). The GenomeLab GeXP Start Kit was purchased from Beckman Coulter, Inc. (Miami, FL, USA), and the Total RNA Isolation Kit was purchased from RBC Bioscience Corp. (Taipei, Taiwan). Magnesium chloride (MgCl_2_), DNA Taq polymerase, HPLC grade water, acetic acid, acetonitrile, methanol, and isopropanol were purchased from Thermo Fisher Scientific (Pittsburgh, PA, USA).

### 2.2. Germination of Brown Rice and Extraction

BR was germinated as reported in an earlier publication [16]. Briefly, BR was sterilized in 0.1% NaOCl (1 : 5, w/v) for 30 min and incubated in 0.5% H_2_O_2_ (1 : 5, w/v) for 6 h. The solvents were discarded and the BR was further incubated at 37°C for another 18 h in anoxic condition. Germination was evidenced by the growth of sprouts. After drying at 50°C, the final moisture content was between 8 and 11%. Dried GBR was ground using a grinder and extracted using ethyl acetate as previously described [23]. Briefly, GBR powder was dissolved in ethyl acetate (1 : 4 w/v). The mixtures were sonicated for 1 h. The extract was filtered through Whatman filter paper number 1 and the entire extraction process was repeated twice on the residue obtained from the filtration process. The filtrates were pooled and solvent was removed from the filtrates under reduced pressure (Rotavapor R210, Buchi, Postfach, Flawil, Switzerland). Finally, the extracts were cooled in a desiccator and kept at −80°C until further analyses.

### 2.3. High Performance Liquid Chromatography (HPLC-DAD)

HPLC analysis for phenolic compounds was performed using Agilent G1310A pumps (Agilent, Santa Clara, CA, USA) with a diode array detector set at wavelengths of 280 nm and 320 nm. Chromatographic separations were performed on a LUNA C-18 column (5 mm, 250 × 4.6 mm) (Phenomenex, Torrance, CA, USA). The solvent composition and gradient elution conditions used were the same as those described previously [17]. The mobile phase was composed of solvent (A) water-acetic acid (94 : 6, v/v, pH 2.27) and solvent (B) acetonitrile. The solvent gradient was 0–15% B for 40 min, 15–45% B for 40 min, and 45–100% B for 10 min. A flow rate of 0.5 mL/min was used and 20 *μ*L of sample was injected. Samples and mobile phases were filtered through a 0.22 *μ*m Millipore filter, type GV (Millipore, Bedford, MA, USA), prior to HPLC injection. Samples were analyzed in triplicate. Determination and quantification of phenolic compounds were done by comparing their retention times and UV-Vis spectral data to known, previously injected standards.

Oryzanol content was analyzed according to Azlan et al. [24] using reverse-phase HPLC. The HPLC system consisted of Hewlett-Packard (Germany) Model G1311A High Performance Liquid Chromatography connected to ALS Autoinjector Series 1100 (Hewlett-Packard, Germany). The mobile phase consisted of acetonitrile/methanol/isopropanol (50 : 45 : 5 by volume). Oryzanol components were detected at 325 nm with a Hewlett-Packard Model 1100 Series Photodiode Array Detector. Oryzanols were separated using a 250 × 4 mm id Hewlett-Packard column packed with 5 mm ODS-C18 Hypersil silica.

### 2.4. Transmission Electron Microscopy (TEM)

Twenty microliters (20 *μ*L) of 20 *μ*M A*β*(1-42) was incubated with or without the presence of 1 ppm and 10 ppm GBR extract for 48 hours at 37°C. The concentrations of GBR extract (1 and 10 ppm) were chosen based on previous determination [23]. The samples were prepared on continuous carbon support films, followed by glow discharging and negative staining of 2% aqueous uranyl acetate (pH 4.5) by the single droplet procedure. The A*β*(1-42) in the presence and absence of GBR extract was adsorbed onto the carbon film for 5 min, dried on a filter paper, and incubated with uranyl acetate solution individually and was viewed under TEM Philips HMG 400 at 10,000x and 20,000x magnifications. A representative assessment of A*β* fibril formation was made at several positions across each EM grid, to avoid any biased or subjective data selection.

### 2.5. Cell Culture

The human neuroblastoma SH-SY5Y cells were grown in complete culture medium containing mixture of Minimum Essential Eagle's Medium and Ham's Nutrient F-12 (1 : 1), which was supplemented with 10% fetal bovine serum, 1% MEM nonessential amino acids, and 50 *μ*g/mL gentamicin. Cells were maintained at 37°C under 5% CO_2_ and 95% air.

### 2.6. MTT Assay

For determination of neuroprotective effects of GBR extract on A*β*(1-42) toxicity, MTT analysis was carried out. SH-SY5Y cells were seeded at a density of 2 × 10^5^ cells/mL in a 96-well plate. Two days after seeding, cells were differentiated with 10 *μ*M retinoic acid for 6 days prior to treatment. Human A*β*(1-42) was diluted to 200 *μ*M and incubated at 37°C for 48 h to produce aggregated A*β* in order to cause toxicity to the cells. Cells were pretreated with GBR extract at 1 ppm and 10 ppm for 24 h before being exposed to aggregated A*β*(1-42) at final concentration of 20 *μ*M for another 24 h prior to MTT analysis.

### 2.7. Lactate Dehydrogenase (LDH) Release Assay

LDH release was determined using CytoTox96 Non-Radioactive Cytotoxicity Assay (Promega, Southampton, UK). In this colorimetric assay, LDH converts lactate and nicotinamide adenine dinucleotide (NAD+) to pyruvate and NADH, respectively. This initial reaction is coupled to a second reaction where diaphorase converts iodonitrotetrazolium salt and NADH to a red colored formazan compound and NAD+, respectively. Cells were seeded at a density of 2 × 10^5^ cells/mL in a 96-well plate. After 2 days of seeding, cells were differentiated with 10 *μ*M retinoic acid for 6 days prior to treatment. Cells were pretreated with GBR extract (1 ppm and 10 ppm) for 24 h prior to treatment with 250 *μ*M H_2_O_2_ for another 2 h [23]. After cells were exposed to H_2_O_2_ with or without pretreatment with GBR, 50 *μ*L of the cell culture medium was transferred from each well of the assay plate to the corresponding well of flat bottom 96-well assay plate. Maximum LDH release was achieved by adding Lysis Buffer to the corresponding wells and 50 *μ*L of the medium was used. Substrate mix was reconstituted using Assay Buffer, from which 50 *μ*L was added to each well of the plate. The plate was covered and incubated at room temperature and protected from light for 30 minutes, after which 50 *μ*L of stop solution was added to each well of plate. Absorbance was read at 490 nm using on BioTek Synergy H1 Hybrid Reader (BioTek Instruments Inc., Winooski, VT, USA). The results were calculated using the formula

(1) % LDH  release=LDH  release  in  the  mediummax.  LDH  release∗100.

### 2.8. DCFH-DA Intracellular ROS Production

Free radical production was measured by incubating the cells with the fluorescent probe 2′,7′-dichlorodihydrofluorescein diacetate (DCFH-DA) according to Rosenkranz et al. [25] with modifications. DCFH-DA freely crosses the cell membranes and it is hydrolyzed by cellular esterases to 2V,7V-dichlorodihydrofluorescein, a nonfluorescent molecule that can be oxidized to the fluorescent 2V,7V-dichlorofluorescein (DCF) in the presence of peroxides. Cells were seeded at a density of 2 × 10^5^ cells/mL in a black 96-well plate and differentiated for 6 days. Prior to treatment with GBR extract, media were discarded and cells were incubated with 100 *μ*L of 10 *μ*M of DCFH-DA for 30 minutes. DCFH-DA was discarded, and cells were washed twice with PBS and pretreated with GBR extract for 24 h. Then, 250 *μ*M H_2_O_2_ was added to respective wells and accumulation of DCF in the cells was measured as an increase in fluorescence (480 nm excitation, 510 nm emission) for another 2 h on BioTek Synergy H1 Hybrid Reader (BioTek Instruments Inc., Winooski, VT, USA).

### 2.9. Multiplex Gene Expression Analysis

#### 2.9.1. Primer Design

Primers were designed on the GenomeLab eXpress Profiler software using* Homo sapiens* sequence adopted from the National Center for Biotechnology Information GenBank Database. The genes of interest, housekeeping genes, and internal control are shown in Table 1. The forward and reverse primers had universal tag sequences in addition to nucleotides that were complementary to the target genes. Primers were supplied by First Base Ltd. (Selangor, Malaysia) and diluted in 1x TE Buffer to a concentration of 500 nM for reverse primers and 200 nM for forward primers.

#### 2.9.2. Extraction of RNA

Cells were seeded at a density of 2 × 10^5^ cells/mL in 6-well plate. After 2 days of seeding, cells were differentiated with 10 *μ*M retinoic acid for 6 days. Cells were pretreated with GBR extract for 24 h prior to treatment with 250 *μ*M H_2_O_2_ for another 2 h. Treated SH-SY5Y cells were washed with phosphate-buffered saline (PBS), and RNA was isolated using Total RNA Isolation Kit (RBC Bioscience Corp., Taiwan) according to manufacturer's instructions. RNA concentration was determined using NanoDrop spectrophotometer (Thermo Scientific NanoDrop, NanoDrop Technologies, Wilmington, DE, USA). The ratios of A260/230 and A260/280 were used to indicate the purity of extracted total RNA.

#### 2.9.3. Reverse Transcription and Polymerase Chain Reaction (PCR)

Reverse transcription (RT) and multiplex PCR of RNA samples (50 ng/*μ*L) were carried out in XP Thermal Cycler (BIOER Technology, Hangzhou, China) according to the GenomeLab GeXP Start Kit protocol (Beckman Coulter, Inc., Miami, FL, USA). Briefly, RT reaction mixture was prepared using RNA sample (1 *μ*L each), 4 *μ*L of 5x RT Buffer, 2 *μ*L of RT reverse primers, 1 *μ*L of KanR, 1 *μ*L of reverse transcriptase, and 11 *μ*L of DNAse/RNase-free water. cDNA was synthesized according to the reaction protocol: 48°C for 1 min, 42°C for 60 min, 95°C for 5 min, and 4°C hold. Then, 9.3 *μ*L of each RT product was mixed with 10.7 *μ*L of PCR reaction mixture consisting of 5x PCR Buffer, 25 mM MgCl_2_, PCR forward primer plex, and Thermo-Start DNA polymerase. Amplification conditions were 95°C for 10 min, followed by 34 cycles of 94°C for 30 sec, 55°C for 30 sec, 70°C for 1 min, and 4°C hold.

#### 2.9.4. GeXP Data Analysis

PCR products (1 *μ*L each) from the above reactions were mixed with 38.5 *μ*L of sample loading solution and 0.5 *μ*L of DNA size standard 400 (Beckman Coulter, Inc., Miami, FL, USA) in a 96-well sample loading plate and analyzed on the GeXP machine (Beckman Coulter, Inc., Miami, FL, USA). The results from the machine were analyzed using the Fragment Analysis module of the GeXP system software and then imported onto the analysis module of eXpress Profiler software. Normalization was performed with EEF1A1 according to manufacturer's instructions.

### 2.10. Statistical Analysis

Data were analyzed by using SPSS (Statistical Program for the Social Sciences version 20, SPSS Inc., USA). All experiments were carried out in triplicate. The results were expressed as mean ± SD. Analyses of variances (ANOVA) were carried out to evaluate the significant difference, and Tukey tests were used to determine the significance of the difference. Linear correlation was determined using Pearson correlation, and *p* < 0.05 was considered significant.

## 3. Results and Discussion

### 3.1. HPLC Determination of GBR Bioactive Compounds

The germination process of BR is known to affect its composition and functional properties [26]. The improved concentration of several bioactive compounds present in GBR is suggested to be responsible for its health beneficial effects. In the present study, the bioactive compounds shown in Table 2 may have contributed to the bioactivity of the extract. The ethyl acetate extract of GBR was found to contain guaiacol, 2MHQ, rosmarinic acid, cinnamic acid, and *γ*-oryzanols (cycloartenyl ferulate, 24-methylene cycloartanyl ferulate, campesteryl ferulate, and mixtures of *β*-sitosteryl ferulate and cycloartanyl ferulate).

Although other bioactive compounds have been reported in GBR [26], the choice of ethyl acetate in this study may have precluded their extraction. However, the presence of multiple bioactive compounds in the ethyl acetate extract may contribute to synergistic improvements in bioactivity. Moreover, the bioactivity of GBR is reported to be due to the presence of multiple bioactive compounds through the process of food synergy, and as the data here demonstrates, any bioactivity observed in the present study would be attributed to the presence of multiple compounds, as we have demonstrated severally [27]. As can be recalled, oxidative stress is strongly implicated in neuronal cell death in AD [14]. Furthermore, the bioactive compounds in the ethyl acetate extract of GBR are mostly phenolics, which have been shown to have potent antioxidant effects, and as such can counter the effects of oxidative stress [28].

### 3.2. Antiamyloidogenic Properties of GBR

**Figure 1:**
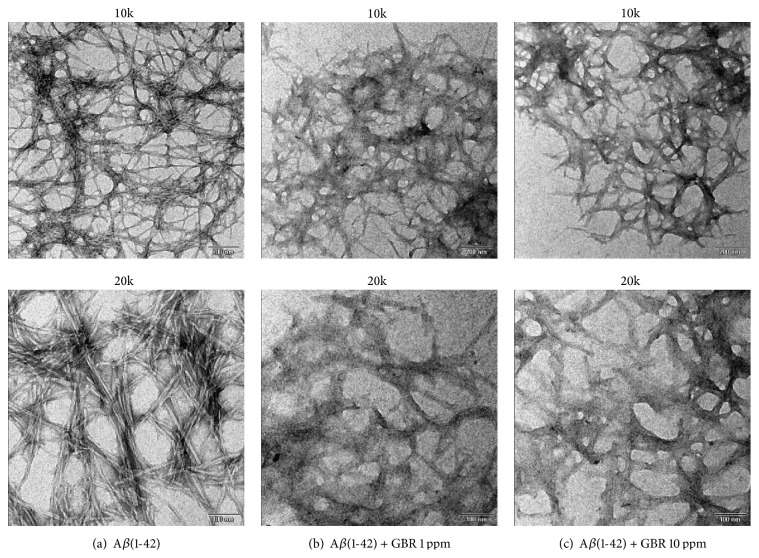
Antiamyloidogenic properties of GBR. (a) 20 *μ*M A*β*(1-42) incubated for 48 h at 37°C under near-physiological conditions assembled into negatively stained amyloid fibrils. (b and c) 20 *μ*M A*β*(1-42) in the presence of 1 ppm and 10 ppm of GBR, respectively, incubated for 48 h at 37°C under near-physiological conditions formed amorphous positively stained deposits showing remnants of a fibril-like morphology. TEM images with 10kx magnification with scale bar = 200 nm and 20kx magnification with scale bar = 100 nm.

Due to the limited capability of neuron cells to regenerate, it is vital to prevent cell death through regulation of oxidative stress and *β*-amyloid production, formation, fibrillogenesis, and deposition in order to prevent their damaging effects on brain cells. As such, compounds or drugs that are able to regulate these processes have huge potentials in the management of AD. Accordingly, anti-inflammatory drugs [29] and antioxidative compounds [30] have been shown to prevent and slow down the onset of AD, through possessing antiamyloidogenic properties. Additionally, recent studies have shown that epigallocatechin gallate (EGCG) [31] and cannabinoids [32] can reduce the formation of A*β* fibrils and aggregates formation, while a hybrid of the AD drug tacrine, tacrine-6-ferulic acid dimer, was reported to alter the conformation of A*β* and block A*β*-induced cell death as its possible neuroprotective action [33]. In this study, ethyl acetate extract of GBR also altered A*β* formation (Figure 1). In the absence of GBR extract, A*β*(1-42) appeared negatively stained and showed characteristic fibrillary amyloid morphology. However, in the presence of GBR (1 and 10 ppm), A*β*(1-42) showed amorphous positively stained deposits, resembling remnants of amyloid fibril morphology [34]. In fact, at 10 ppm of GBR, A*β*(1-42) formed distinctly amorphous deposits that appeared very different from those present when GBR was not added. This is consistent with findings on the ability of natural product compounds to inhibit A*β* aggregation [15].

### 3.3. GBR Attenuated A*β*(1-42)-Induced Cell Death

**Figure 2:**
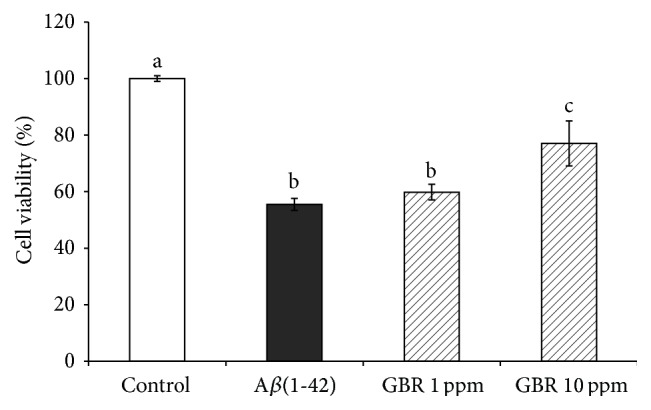
MTT analysis on SH-SY5Y cells. Cells were pretreated with GBR extracts at 1 ppm and 10 ppm individually for 24 h and subsequently incubated with or without A*β*(1-42) for 24 h. Results are expressed as mean ± SD, different letters representing different groups indicating significant difference (*p* < 0.05).

GBR extract ameliorated the aggregation of A*β*(1-42); thus we further assessed its protective effects on A*β*(1-42) toxicity in SH-SY5Y cells. In this study, we incubated aggregated A*β*(1-42) with or without the presence of 1 ppm and 10 ppm of GBR extract.  Figure 2 shows that incubation of SH-SY5Y cells with 20 *μ*M of A*β*(1-42) decreased the cell viability to 55.5% as compared to untreated control. Pretreatment of the cells with GBR extract at 1 ppm and 10 ppm was found to substantially increase the cell survival to 59.82% and 77.02%, respectively. There have been numerous reports on natural compounds with neuroprotective effects against A*β*-induced cell death, many of which exhibited antioxidant properties [30–32]. These antioxidative properties are linked with the regulation of caspase cascade as well as mitogen activated protein kinases (MAPKs) [35, 36]. Moreover, GBR extract used in this study has been previously reported to regulate cell survival and apoptosis pathway through the modulation of MAPKs (JNK, ERK, and p38), p53, AKT, and NF-*κβ* [23].

### 3.4. GBR Attenuated H_2_O_2_-Induced Neurotoxicity

**Figure 3:**
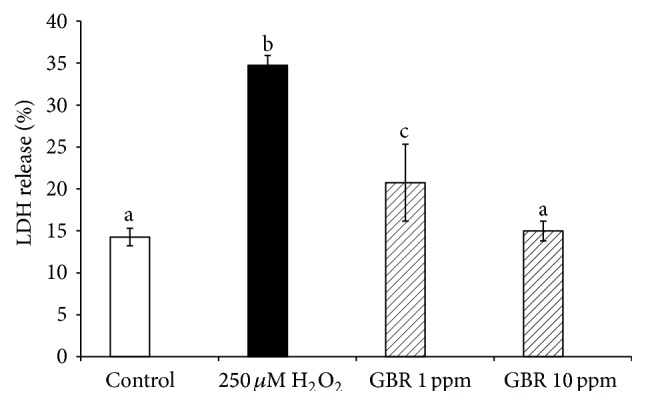
Lactate dehydrogenase release from SH-SY5Y cells. Cells were pretreated with GBR extracts at 1 ppm and 10 ppm individually for 24 h and subsequently incubated with or without 250 *μ*M H_2_O_2_ for 2 h. Results are expressed as mean ± SD, different letters representing different groups indicating significant difference (*p* < 0.05).

To study the neuroprotective effects of GBR on oxidative stress-induced toxicity in SH-SY5Y cells, the cells were pretreated with GBR at 1 and 10 ppm for 24 h prior to H_2_O_2_ insult.  Figure 3 shows that GBR reduced the release of LDH from the cells, which may have indicated the amelioration of oxidative stress-induced neurotoxicity in differentiated SH-SY5Y neuroblastoma cells in comparison to nontreated cells. LDH is a stable cytoplasmic enzyme which is released from the cells due to loss of plasma membrane integrity. In an* in vitro* model of cell apoptosis, an increase in LDH activity in the culture supernatant signifies an increase in dead cells or disintegration of plasma membrane. Thus, the measurement of cytosolic LDH release from the cells is commonly used to indicate the loss of membrane integrity especially in apoptotic cells [37].

**Figure 4:**
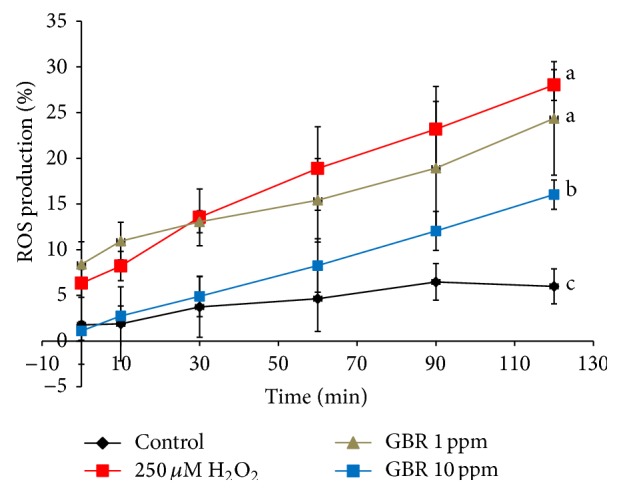
Intracellular reactive oxygen species (ROS) generation in SH-SY5Y cells. Cells were pretreated with GBR extracts at 1 ppm and 10 ppm individually for 24 h and subsequently incubated with or without 250 *μ*M H_2_O_2_ for 2 h. Results are expressed as mean ± SD, different letters representing different groups indicating significant difference (*p* < 0.05).

From the determination of ROS production using DCFH-DA as represented by Figure 4, it was observed that H_2_O_2_ increased the level of intracellular ROS throughout the incubation time in comparison to the untreated normal control cells. Cells pretreated with GBR prior to the addition of H_2_O_2_, nevertheless, exhibited lower levels of ROS, in corroboration of the GBR-induced attenuation of neurotoxicity as evidenced by reduced LDH release. This is in agreement with our previous findings by which an ethyl acetate extract of GBR possessed high antioxidative capacity and regulated several antioxidant genes such as superoxide dismutases (SODs) and catalase [23]. Oxidative stress results from imbalance between the levels of antioxidants and prooxidants and has been the target in the management of many chronic diseases. In the brain, increased levels of oxidative damage are implicated in aging as well as the processes leading up to neurodegenerative diseases like AD. The attenuation of ROS by GBR extract suggests its potential for attenuating oxidative stress-induced neurotoxicity.

### 3.5. Transcriptional Regulation of AD-Related Genes by GBR

Oxidative stress plays vital roles in the development of A*β* peptides through the regulation of APP metabolism [10, 14]. The amyloid-cascade theory hypothesizes that proteolytic cleavage of APP may result in neurotoxic *β*-amyloid peptides, which are the hallmark of AD [2]. However, as can be recalled, depending on the site of cleavage of the APP molecule by *α*-, *β*-, or *γ*-secretases, the metabolic processing of APP may be in favor of amyloidogenic or nonamyloidogenic pathways [3]. Unlike in the nonamyloidogenic pathway which precludes the formation of neurotoxic A*β*, the amyloidogenic pathway produces aggregated, fibrillary complexes termed senile plaques, which were found to be abundant in AD [38].

**Figure 5:**
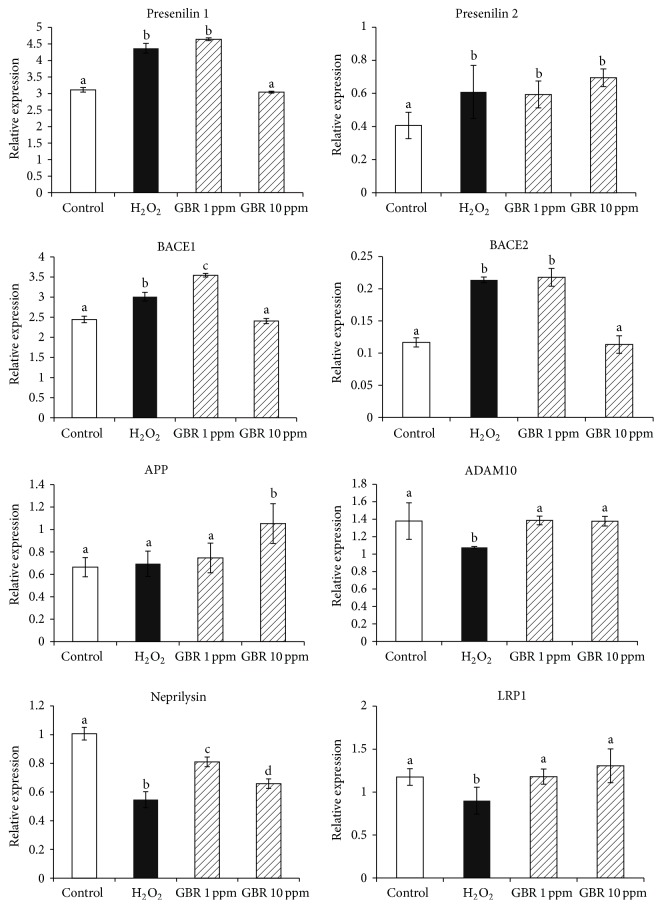
mRNA levels of Alzheimer's disease-related genes in differentiated SH-SY5Y neuroblastoma cells induced with 250 *μ*M hydrogen peroxide (H_2_O_2_), with or without pretreatment with germinated brown rice (GBR) at 1 ppm and 10 ppm, respectively. Different letters on bars representing different groups indicating significant difference (*p* < 0.05). ADAM10: a disintegrin and metalloproteinase domain-containing protein 10; APP: amyloid precursor protein; BACE: beta-site APP-cleaving enzyme; LRP: low density lipoprotein receptor-related protein.

In the present study, we analyzed the effects of H_2_O_2_ on AD-related genes as well as the ability of GBR to modulate their transcription (Figure 5). Expression levels of Presenilin 1, Presenilin 2, A*β* cleaving enzymes (BACE1, BACE2), ADAM10, LRP, Neprilysin, and APP were determined. Presenilin 1, Presenilin 2, BACE1, and BACE2 were found to be upregulated as a result of H_2_O_2_-induced oxidative stress in comparison with the untreated normal cells. Furthermore, pretreatment with GBR attenuated the H_2_O_2_-induced transcriptional changes on Presenilin 1, BACE1, and BACE2, but not that of Presenilin 2. Presenilin 1 and Presenilin 2 are important *γ*-secretase enzymes with protease activities that cleave APP to produce A*β* fragments, and their deficiency or inhibition may reduce A*β* production [39]. This has been suggested as a potential target for antiamyloidogenic approach in AD research [40]. On the other hand, BACE1 and its homolog BACE2 form part of the *β*-secretases that are involved in the processing of APP to produce A*β* [41]. BACE2 was reported to be responsible for the release of A*β* in mutant transfected cells [42] and was able to cleave APP at the secretase cleavage site, in an* in vitro* model [43]. BACE1, on the other hand, has been established as a more dominant inducer of APP cleavage leading to A*β* formation [42, 44]. Moreover, in a transgenic mice model bearing APP mutation, overexpression of BACE increased the processing of APP and contributed to high levels of A*β* [45]. This is consistent with findings of elevated BACE activity in the brains of AD patients with high load of A*β* plaques [46].

Although accumulated A*β* can be toxic, the sAPP*α* is protective towards neuronal cells. In the present study, H_2_O_2_-induced oxidative stress did not promote APP expression in comparison to the normal control (*p* > 0.05). Interestingly, we found that pretreatment with GBR (10 ppm) upregulated APP expression. There are reports of the neuroprotective effects of soluble APP mediated by *α*-secretase activity in PC12 cells [47]. Our study also showed that the expression of ADAM10, a part of *α*-secretase, was downregulated under oxidative stress condition, while GBR increased its expression similar to what was observed in the normal control. This is in agreement with previous findings that support the neuroprotective effects of ADAM10 [48], thus suggesting the potential role of *α*-secretases, particularly ADAM10 in the therapeutic development of AD.

Similarly, H_2_O_2_ treatment resulted in reduced expression of Neprilysin and LRP genes when compared to the control (*p* < 0.05). Treating the cells with GBR prior to the H_2_O_2_ insult, nevertheless, resulted in an increased expression of Neprilysin and LRP genes. Moreover, the expression of LRP in GBR-treated cells was found to be dose-dependent, implying that GBR is able to increase the transport of A*β* from the brain into the bloodstream. Aside from its involvement in cholesterol metabolism and transport, LRP binds several ligands including ApoE, APP, and A*β* [49] and has been demonstrated to regulate the clearance of A*β* from the brain into the bloodstream [50]. Accordingly, A*β* transport clearance across the blood-brain barrier (BBB) in mice was reported to decrease with aging [51], the basis of which may be the loss of LRP transport and hence the accumulation of A*β* in blood vessels and perivascular brain tissue, as demonstrated in aged individuals [52]. Similarly, brain vascular dysfunction, reduced cerebral blood flow, BBB leakage, and accumulation of multiple blood-derived vasculotoxins and neurotoxins following injury to brain endothelial cells have recently been shown to collectively contribute to AD pathogenesis [53]. Moreover, A*β* binds to brain endothelial LRP1 at the abluminal side of the BBB and clears into the peripheral circulation [54] and has been demonstrated to induce oxidative stress and mediates BBB changes and brain capillary dysfunction [55]. Conversely, antioxidants in GBR extract may protect brain vasculature and improve LRP1 mediated clearance of A*β*, thereby attenuating the effects of A*β*-induced toxicity. On the other hand, Neprilysin is reported to contribute towards the degradation of A*β* [56]. In the hippocampal region of AD patients, it was demonstrated that Neprilysin mRNA and protein levels were significantly lower than in control subjects [57]. Accordingly, deficiency in Neprilysin may result in limited degradation of both endogenously produced A*β* and exogenously administered A*β* [58] and could eventually promote the accumulation of A*β* in AD. Thus, regulation of LRP and Neprilysin genes by GBR suggested that the neuroprotective effects of GBR in reducing the accumulation of A*β* are partly mediated by increased degradation and clearance of A*β* from the brain tissue.

**Figure 6:**
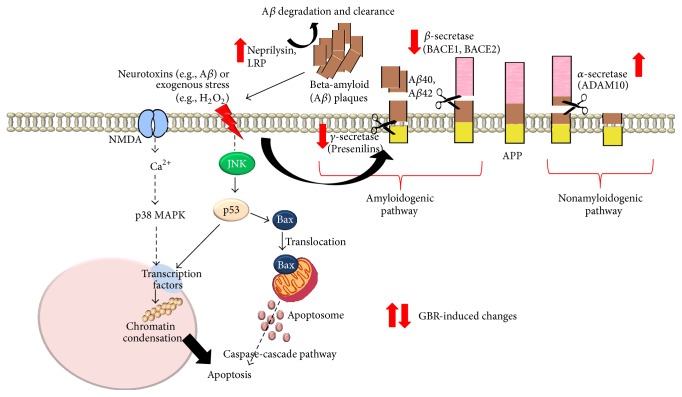
Proposed schematic diagram showing targets of germinated brown rice (GBR) in APP processing metabolism pathway. ADAM10: a disintegrin and metalloproteinase domain-containing protein 10; APP: amyloid precursor protein; BACE: beta-site APP-cleaving enzyme; Bax: BCL2-associated X protein; H_2_O_2_: hydrogen peroxide; JNK: c-Jun N-terminal kinases; LRP: low density lipoprotein receptor-related protein; p53: p53 tumor suppressor.

Based on the transcriptional regulation of AD-related genes by GBR, in neuronal cells that have been exposed to H_2_O_2_, we propose that the mechanistic basis for the neuroprotective effects of GBR may be through multiple pathways (Figure 6) including the regulation of APP metabolism genes as well as apoptosis pathway involving JNK, ERK, and p53, as we have previously demonstrated [23]. The antiamyloidogenic effects of GBR may be due to its bioactive-rich content with potent antioxidant effects that are able to counter H_2_O_2_-induced neurotoxicity.

## 4. Conclusion

In the present study, we demonstrated that GBR modified the structure of A*β*(1-42) when incubated together and also protected neuronal cells from H_2_O_2_-induced neurotoxicity (reduced LDH release) partly through reduced oxidative stress (lowered ROS production). We also showed that transcriptional regulation of AD-related genes by GBR tended towards neuroprotection. As such, we propose that GBR not only possesses the potential to manage oxidative stress and oxidative stress-induced A*β* formation, but also can reduce the aggregation of A*β* as well as increase its clearance and degradation. Further work involving* in vivo* model to assess the effectiveness of GBR at physiological level is of importance and is therefore indicated.

## Figures and Tables

**Table 1:** Gene names, accession numbers, and reverse and forward primer sequences used in GeXP Multiplex Gene Expression Analysis.

Gene name	Accession number	Primer sequences^*∗*^ with universal tags (underlined)	
		Forward	Reverse
ACTB^a^	NM_001101	AGGTGACACTATAGAATAGATCATTGCTCCTCCTGAGC	GTACGACTCACTATAGGGAAAAGCCATGCCAATCTCATC
ADAM10	NM_001110	AGGTGACACTATAGAATACTAAACCAAACTTCACAGACT	GTACGACTCACTATAGGGATTCTTCATACAGCATACATGG
APP	NM_000484	AGGTGACACTATAGAATAGGTTTGGCACTGCTC	GTACGACTCACTATAGGGACATTCATGTGCATGTTC
BACE1	NM_001207048	AGGTGACACTATAGAATATTGAAGCTGCAGTCAAAT	GTACGACTCACTATAGGGAATGGCAAACTTGTAACAGTC
BACE2	NM_012105	AGGTGACACTATAGAATAGGCCTGAATTATGAATGTTA	GTACGACTCACTATAGGGAGCATAGGACACAATCCAC
EEF1A1^a,#^	NM_001402	AGGTGACACTATAGAATACACACGGCTCACATTGCAT	GTACGACTCACTATAGGGACACGAACAGCAAAGCGA
GAPDH^a^	NM_002046	AGGTGACACTATAGAATAAAGGTGAAGGTCGGAGTCAA	GTACGACTCACTATAGGGAGATCTCGCTCCTGGAAGATG
Glucuronidase^a^	NM_000181	AGGTGACACTATAGAATAGGTTGGAGAGCTCATTTGGA	GTACGACTCACTATAGGGAGAACAGTCCAGGAGGCACTT
KanR^b^	—	AGGTGACACTATAGAATAATCATCAGCATTGCATTCGATTCCTGTTTG	GTACGACTCACTATAGGGAATTCCGACTCGTCCAACATC
LRP1	NM_002332	AGGTGACACTATAGAATAATCGATCTTCACAAAGGAG	GTACGACTCACTATAGGGAATCTGATCCAGGTTACTCTC
Neprilysin	NM_000902	AGGTGACACTATAGAATACCTTAGCAATTAAAGAAAGG	GTACGACTCACTATAGGGAGTTGTTTACTTTGGCTGAAT
Presenilin 1	NM_000021	AGGTGACACTATAGAATATACTTCCAGAATGCACAGA	GTACGACTCACTATAGGGATTCTTCCTCATCTTGCTC
Presenilin 2	NM_000447	AGGTGACACTATAGAATAGGAAGAAGTGTGTGATGAG	GTACGACTCACTATAGGGAGTATTTGAGGGTCAGCTCT

^*∗*^Based on the *Homo sapiens* gene sequences adopted from the National Center for Biotechnology Information GenBank Database. ^a^Housekeeping genes; ^b^internal control; ^#^normalization gene. ACTB: beta-actin; ADAM: a disintegrin and metalloproteinase; APP: amyloid precursor protein; BACE: beta-site APP-cleaving enzyme; EEF1A1: eukaryotic translation elongation factor 1 alpha 1; GAPDH: glyceraldehyde 3-phosphate dehydrogenase; LRP1: low density lipoprotein receptor-related protein 1.

**Table 2:** Bioactive compounds in ethyl acetate extract of germinated brown rice as determined by HPLC-DAD.

Compounds	Concentration (mg/g extract)
Guaiacol	0.023 ± 0.04
2MHQ	0.947 ± 0.03
Rosmarinic acid	0.046 ± 0.04
Cinnamic acid	0.654 ± 0.02
Cycloartenyl ferulate	2.219 ± 0.32
24-Methylene cycloartanyl ferulate	5.35 ± 0.67
Campesteryl ferulate	2.74 ± 0.74
Mixtures of *β*-sitosteryl ferulate and cycloartanyl ferulate	6.30 ± 0.10

Values represent mean ± SD (*n* = 3).

## Abbreviations

AD: Alzheimer's disease
A*β*: Beta-amyloid
H_2_O_2_: Hydrogen peroxide
GBR: Germinated brown rice
APP: Amyloid precursor protein beta-site
BACE: Beta-site APP-cleaving enzyme
ADAM10: A disintegrin and metalloproteinase domain-containing protein 10
LRP: Low density lipoprotein receptor-related protein
ROS: Reactive oxygen species
BR: Brown rice
PBS: Phosphate-buffered saline
NaOCl: Sodium hypochlorite
DCFH-DA: Dichlorodihydrofluorescein diacetate
DCF: 2V,7V-Dichlorofluorescein
TEM: Transmission Electron Microscopy
LDH: Lactate dehydrogenase
HPLC: High performance liquid chromatography
SOD: Superoxide dismutase
ACTB: Beta-actin
GAPDH: Glyceraldehyde 3-phosphate dehydrogenase.
